# Suitability of invasive gobies as paratenic hosts for acanthocephalans of the genus *Pomphorhynchus* sp.

**DOI:** 10.1017/S0031182024001197

**Published:** 2024-12

**Authors:** Milen Nachev, Michael Hohenadler, Nicklas Bröckers, Daniel Grabner, Bernd Sures

**Affiliations:** 1 Department of Aquatic Ecology, University of Duisburg-Essen, Essen, Germany; 2 Centre for Water and Environmental Research (ZWU), University of Duisburg-Essen, Essen, Germany; 3 Research Center One Health Ruhr, Research Alliance Ruhr, University of Duisburg-Essen, Essen, Germany; 4 Water Research Group, Unit for Environmental Sciences and Management, North-West University, Potchefstroom, South Africa

**Keywords:** invasive gobies, paratenic host, *Pomphorhynchus* sp.

## Abstract

Ponto-Caspian gobies became highly abundant in many regions outside their native distribution range (e.g. in the Rhine River system). In the newly invaded habitats, the parasite communities of the invasive gobies are characterized by a lower species richness compared to their native range. Interestingly, acanthocephalans of the genus *Pomphorhynchus* are highly abundant, although they do not become mature and mostly remain encapsulated in the abdominal cavity as preadults. Thus, gobiids could either represent a dead-end host for *Pomphorhynchus* sp. declining its population (dilution effect) or act as a paratenic host that could increase the infection pressure if the infected gobies are preyed upon by appropriate definitive hosts (spill back). To determine which of the 2 scenarios the gobiids contribute to, we conducted 2 infection experiments using smaller and larger individuals of the definitive host chub (*Squalius cephalus*), infected with preadults of *Pomphorhynchus* sp. collected from the abdominal cavity of *Neogobius melanostomus*. The results showed that preadults were able to complete their development and mature in the definitive host with mean recovery rates of 17.9% in smaller and 27.0% in larger chubs. Successful infections were observed in 62.0% and 80.0% of the smaller and larger chubs, respectively. Our study demonstrated that gobies can theoretically serve as a paratenic host for acanthocephalans of the genus *Pomphorhynchus*, and that infection might spill back into the local fish community if infected gobies are preyed upon by suitable definitive hosts of *Pomphorhynchus* sp. such as large barbel or chub.

## Graphical abstract

**Figure:**
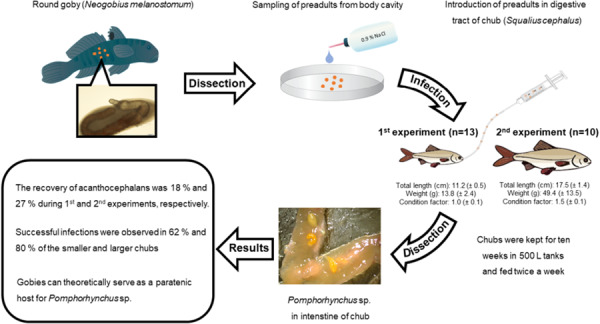

## Introduction

Invasive free-living species may directly and indirectly influence the biodiversity in newly invaded habitats after their establishment. Their impact is mostly attributed to direct interaction with the local free-living communities (e.g. competition for resources, predation, hybridization with native species etc.) (Mooney and Cleland, 2001; Dextrase and Mandrak, 2006; Shochat *et al*., 2010; Havel *et al*., 2015). An indirect mode of action could be due to changes in the composition of the parasite communities in the new area (Torchin *et al*., 2003; Calhoun *et al*., 2018; Hohenadler *et al*., 2019), as parasites can reduce host density (Anderson and May, 1978; May and Anderson, 1978; Kuris and Lafferty, 1992; Hudson *et al*., 1998) or decrease host body size (Torchin *et al*., 2001). For example, invasive species can co-introduce their own endemic parasites, which can **spill over** to native host populations (Torchin *et al*., 2003; Prenter *et al*., 2004; Kelly *et al*., 2009; David *et al*., 2018; Hohenadler *et al*., 2018*a*). Additionally, invasive species can contribute to the life cycle of native parasites. If an invasive species can serve as a suitable host (e.g. intermediate, paratenic or final host) for local parasites, these parasites may **spill back** to other local hosts, thereby increasing their infection rates within the native host populations (Kelly *et al*., 2009; Šlapanský *et al*., 2016). In contrast, invasive species might also be responsible for a decrease of the infection risk in the native host populations (e.g. Gagne *et al*., 2016; Šlapanský *et al*., 2016).This so-called **dilution effect** occurs if the invaders serve as inappropriate hosts for local parasites, in which the parasites cannot develop further or if they are not favoured as food item by a predatory definitive host (Ostfeld and Keesing, 2000; Johnson *et al*., 2012).

Ponto-Caspian gobies (Osteichthyes, Gobiidae) have a high invasion potential, which allowed them to spread into areas distant from their native range. In recent years, they successfully established in the Baltic, Aegean and North Sea basins (Skóra and Stolarski, 1993; Kakareko *et al*., 2009; Mierzejewska *et al*., 2011; Herlevi *et al*., 2017) and even in the North American Great Lakes (Corkum *et al*., 2004; Kornis *et al*., 2013). After the inauguration of the Maine-Danube canal in 1992 several Ponto-Caspian gobiids have invaded the Rhine River system (Stemmer, 2008; van Kessel *et al*., 2009; Borcherding *et al*., 2011; Kalchhauser *et al*., 2013), with the round goby *Neogobius melanostomus* and the bighead goby (*Ponticola kessleri*) being the most abundant and widespread species (Kottelat and Freyhof, 2007; Borcherding *et al*., 2011). Although some ecological parameters such as density, fecundity, growth, predation and parasitism were already studied in gobies from several non-native regions of the Danube River and Rhine River (Kvach, 2002; Jurajda *et al*., 2005; Adámek *et al*., 2007; L'avrinčíková and Kováč, 2007; Kvach and Stepien, 2008; Kováč *et al*., 2009; Ondračková *et al*., 2009; Mühlegger *et al*., 2010; Kalchhauser *et al*., 2013; David *et al*., 2018), their impact on species assemblages in the ecosystems throughout Europe and the River Rhine in particular still remains largely unknown. Ponto-Caspian gobies for example were found to negatively affect the population densities of some native fish species (Dubs and Corkum, 1996; Mooney and Cleland, 2001; Balshine *et al*., 2005; Karlson *et al*., 2007; Jakšić *et al*., 2016; van Kessel *et al*., 2016).

According to previous studies more than 20 different parasite species are known to infest gobiids in the native range of their distribution (e.g. Lower Danube and Black Sea area; see Kvach, 2005; Ondračková *et al*., 2006), while a significantly lower number of species is usually reported for non-native areas. Within the well documented and studied Rhine River for example, only 8 different species have been reported for *N. melanosomus* (Emde *et al*., 2012, 2014; Ondračková *et al*., 2015) and 7 for *P. kessleri* (Ondračková *et al*., 2015). In general, Ponto-Caspian gobies show high infestation rates with acanthocephalans of the genus *Pomphorhynchus* in both their native and non-native range, with a prevalence often exceeding 90% and correspondingly high intensities (Kvach and Skóra, 2006; Francová *et al*., 2011; Emde *et al*., 2014; Ondračková *et al*., 2015). Hohenadler *et al*. (2018*a*) provided examples of how the acanthocephalan *Pomphorhynchus laevis* (now most likely to be considered as *Pomphorhynchus bosniacus*, see Reier *et al*., 2019), which was introduced by Ponto-Caspian invaders (spill-over), can outcompete a local acanthocephalan species (*Pomphorhynchus tereticollis*) and thus change the species composition of the parasite communities in the Rhine system. As the evidence for the occurrence of *P. bosniacus* in Central Europe (Reier *et al*., 2019) was published after the study by Hohenadler *et al*. (2018*a*), it cannot be decided with certainty in retrospect whether they found *P. laevis* or *P. bosniacus* in the Rhine system although the latter seems to be more likely.

However, individuals of *Pomphorhynchus* sp. cannot complete their life cycle in gobies and therefore remain encysted in their abdominal cavity as larval or preadult stages. Mainly cyprinids serve as appropriate definitive hosts, with fish species such as barbel (*Barbus barbus*), chub (*Squalius cephalus*) or idle (*Leuciscus idus*) playing the major roles in the River Rhine (David *et al*., 2018; Hohenadler *et al*., 2018*b*). Until now, the relevance of Ponto-Caspian gobiids for the transmission of *Pomphorhynchus* spp. remains unclear. On the one hand, they can reduce the risk of infection for the native fish populations (dilution effect) if the acanthocephalans cannot be transmitted successfully from gobiids to other fish hosts. On the other hand, the gobiids might increase the infection risk, if they serve as appropriate paratenic hosts (spill back), which are preyed upon by local piscivorous definitive hosts (see e.g. Grabowska *et al*., 2023). A similar case was already demonstrated for larvae of *Anguillicola crassus* detected in gobies from the Rhine River that remain infectious for the final host, the European eel (Hohenadler *et al*., 2018*b*).

The aim of this study was to investigate whether the preadult *Pomphorhynchus* sp. detected in invasive gobies remain infectious to the final host, and whether gobies can thus theoretically contribute to the transmission of these acanthocephalans to their final hosts. To evaluate the infection potential of preadult (larval) stages obtained from the abdominal cavity of gobiids, a laboratory infection experiment with chub (*S. cephalus*) was performed to determine infection success (i.e. recovery rate) and development of the preadult acanthocephalans. The resulting data were compared with results from previous infection experiments (Siddall and Sures, 1998; Sures and Siddall, 1999, 2003; Sures *et al*., 2003; Ruchter, 2012; Le *et al*., 2016, 2018), in which cystacanths of *Pomphorhynchus* sp. obtained from the amphipod intermediate host were administered to chub.

## Materials and methods

### Acanthocephalan sampling and fish infection

Infection experiments with acanthocephalans of the genus *Pomphorhynchus* were performed with chub (*S. cephalus*), as an appropriate definitive host. The fish were obtained from aquaculture facilities of the Research Institute for Nature and Forest, Belgium, where they were raised in spring water. Accordingly, they could be assumed to be free of any infections with metazoan parasites, which was verified by ten randomly chosen chub, which were killed, dissected and screened with light microscopy for parasites.

**Figure 1.**
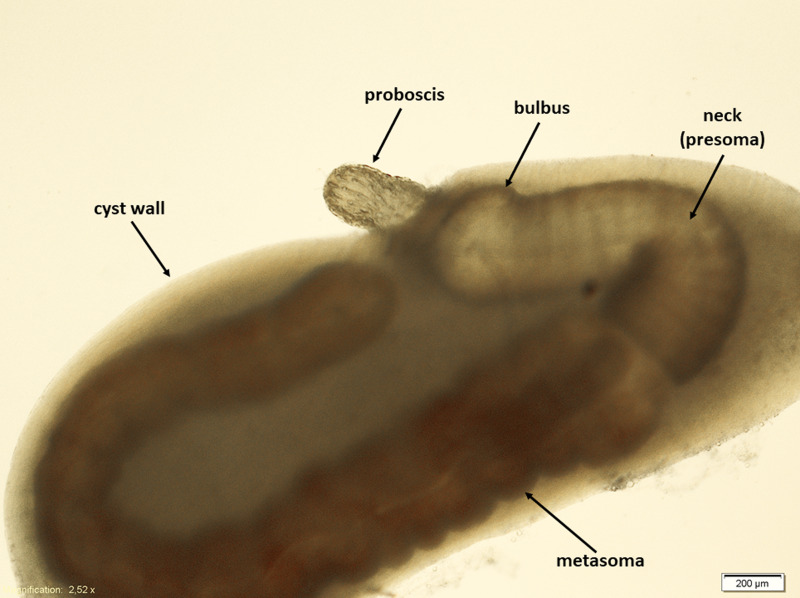
General occurrence of preadult *Pomphorhynchus* sp. obtained from the abdominal cavity of gobiids.

Cysts containing preadults of *Pomphorhynchus* sp. were collected from the abdominal cavity of *N. melanostomus*. The latter were sampled in March and October 2016 by professional fishermen near Kalkar at the Lower Rhine River (844 river km) and kept alive in aerated water tanks. Prior to the infection experiments, the gobies were sacrificed, dissected and the extracted acanthocephalans were placed in physiological saline (0.9% NaCl) and stored at 5°C overnight. Most of the preadult acanthocephalans were entirely enclosed by a fibrous capsule of variable thickness (see Fig. 1). Worth noticing here is, that some thick-walled cysts were not light transparent at all and the preadults inside appeared almost completely degenerated. Therefore, such cysts with preadults were not considered for the infection experiments.

Subsequently, the cysts containing the larvae were collected randomly from a Petri-dish and were introduced to the digestive tracts of chubs by using a 2 ml syringe equipped with a 12 cm plastic tubing of 1 mm diameter (for details see Sures and Siddall, 2003). In total, 23 chubs were experimentally infected, where 1 group of fish (*n* = 13) was infected with 6 acanthocephalans per fish (1^st^ experiment) and another group of fish (*n* = 10) was infected with 10 acanthocephalans each (2^nd^ experiment). As both groups were not infected at the same time (approximately 6 months difference) the second group of chubs (*n* = 13) exhibited on average a larger body size than the other (17.4 cm total *vs* 11.2 cm length, respectively; see Table 1). However, chubs were from the same brood and were held under the same conditions over time. Before and after the infection, the fish were kept at approximately 20°C water temperature in 500 l tanks with dechlorinated tap water and fed twice per week with commercial pellets. A light cycle with a ratio of 16:8 (light: dark) was simulated in order to provide conditions similar to those in their natural habitats. After 10 weeks the chubs were anaesthetized with 150 mg mL^−1^ MS-222 (Merck, Darmstadt, Germany) and then sacrificed by cervical dislocation. After dissection, the parasites were removed from the digestive tracts and counted. All infection experiments were carried out in accordance with the relevant guidelines and regulations and were approved by the Ethics Council (Landesamt für Natur, Umwelt und Verbraucherschutz, Nordrhein-Westfalen, permit number: 84-02.05.40.16.017).

**Table 1.** Morphological data of chub (*S. cephalus*) and mean recovery rates and ranges obtained for chubs infected with preadults (present study) and cystacanths of *Pomphorhynchus* sp. (see Siddall and Sures, 1998; Sures and Siddall, 1999, 2003; Sures *et al*., 2003; Ruchter, 2012; Le *et al*., 2016, 2018)

		Total length (cm)	Weight (g)	Condition factor	Recovery of *Pomphorhynchus* sp. (%)	
Group	*n*	Mean (± s.d.)	Mean (± s.d.)	Mean (± s.d.)	Mean (± s.d.)	Range
Chub (1st experiment)	10	11.2 (± 0.5)	13.8 (± 2.4)	1.0 (± 0.1)	17.9 (± 18.6)	0–50
Chub (2nd experiment)	13	17.5 (± 1.4)	49.4 (± 13.5)	0.9 (± 0.1)	27.0 (± 25.8)	0–70
Siddall and Sures (1998)	21	10.7 (± 7.2)	12.8 (± 2.8)	1.1 (± 0.1)	48.2 (± 25.0)	11–89
Sures and Siddall (1999)	20	10.7 (± 7.7)	13.7 (± 3.3)	1.1 (± 0.2)	44.9 (± 22,6)	10–78
Sures and Siddall (2003)	51	10.9 (± 6.9)	14.5 (± 3.0)	1.2 (± 0.1)	50.8 (± 24.5)	11–100
Sures *et al*. (2003)	36	11.3 (± 1.0)	10.6 (± 3.3)	0.8 (± 0.1)	70.9 (± 20.2)	30–100
Ruchter (2012)	55	10.9 (± 0.9)	9.3 (± 2.1)	0.8 (± 0.1)	58.2 (± 25.9)	20–100
Le *et al*. (2016)	14	n.a.	10.7 (± 2.3)	n.a.	53.6 (± 26.8)	0–100
Le *et al*. (2018)	43	11.7 (± 2.8)	17.1 (± 4.2)	0.9 (± 0.1)	65.1 (± 24.3)	0–100

n.a., parameter is not available.

### Molecular identification of parasites

As a recent study published after our infection experiments indicates the presence of *P. bosniacus* in major European rivers (Reier *et al*., 2019), we sequenced random samples of preadults to check the identity of the *Pomphorhynchus* species present in the investigated section of the Rhine River.

DNA was extracted from the preadult *Pomphorhynchus* sp. using a Chelex-protocol. Approximately 0.5 mm pieces of the parasite tissue were cut off and placed in 300 μL of a 10% Chelex 100 resin (Bio-Rad)-solution. Samples were then boiled for 20 min at 95°C and vortexed every 5 min. Subsequently, samples ware cooled on ice and centrifuged at 5,000 ×g for 5 min, whereas the resulting supernatant was used thereafter for PCR. The PCR was conducted with the primers PT/PL COI F and PT/PL COI R according to Tierney *et al*. (2020). The PCR-products were sent for sequencing (Microsynth-Seqlab) and the sequences were compared with the entries for *P. laevis* and *P. bosniacus* in the BOLD database (https://www.boldsystems.org/index.php).

### Calculations and statistical evaluation

Fulton's condition factor (K) was calculated according to Nash *et al*. (2006). The recovery rates were evaluated as a percentage of the acanthocephalans recovered during dissection in relation to the number of administered preadults. In order to compare the infection success of preadult (encapsulated) *Pomphorhynchus* sp. with those of cystacanths, data from our previous infection experiments with cystacanths (Siddall and Sures, 1998; Sures and Siddall, 1999, 2003; Sures *et al*., 2003; Ruchter, 2012; Le *et al*., 2016, 2018), were considered (for details see Table 1).

The metric parameters of fish from the 1st and the 2nd experiment as well as the recovery rates of acanthocephalans were compared with a Mann–Whitney U-test. Kruskal–Wallis test was applied for comparing the current data with studies of Siddall and Sures, 1998; Sures and Siddall, 1999, 2003; Sures *et al*., 2003; Ruchter, 2012; Le *et al*., 2016, 2018. The recovery rates were correlated with condition factors of fish using Spearman rank correlation analysis.

## Results

The subsequent molecular identification of acanthocephalans showed that all sequenced isolates were *P. bosniacus* according to Reier *et al*. (2019), suggesting that the preadults used for the infection experiments can most likely also be classified as *P. bosniacus*.

The metric parameters of fish used in the infection experiment are presented in Table 1. As the infection experiments were performed in different time periods, the sizes of fish individuals between both experimental groups differed significantly (cf. materials and method) with fish from the second infection experiment being significantly larger with respect to total length and body mass. However, the fish condition factor was similar during both experiments (0.97 and 0.91, respectively).

**Figure 2.**
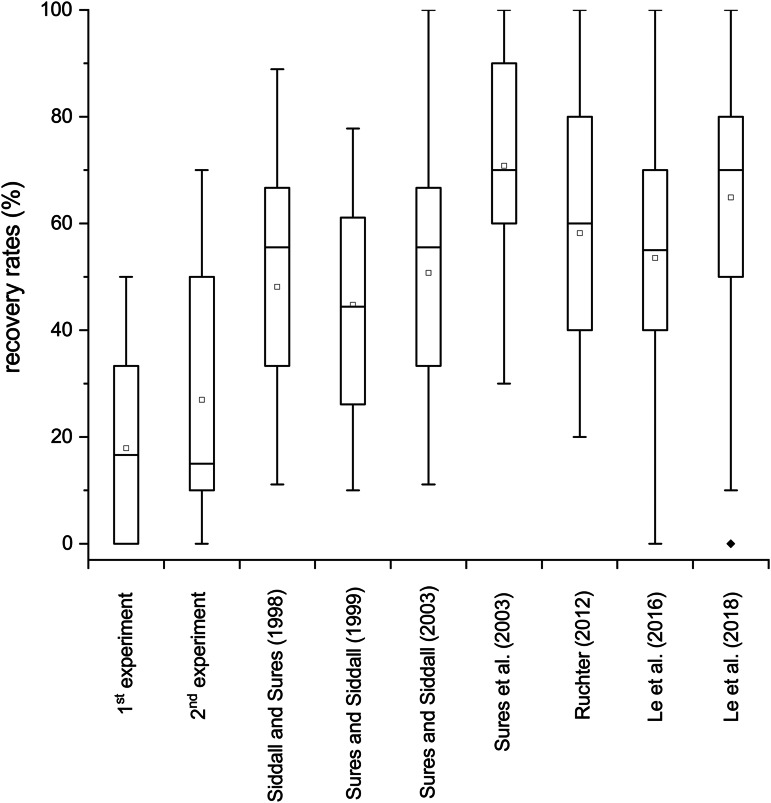
Recovery rates of preadults (current study – 1st and 2nd experiments) and cystacanths of *Pomphorhynchus* sp. (Siddall and Sures, 1998; Sures and Siddall, 1999, 2003; Sures *et al*., 2003; Ruchter, 2012; Le *et al*., 2016, 2018). Open dots are means, lines within the box are medians, boxes are interquartile ranges, error bars are interdecile ranges and closed dots are outliers.

There were no significant differences between recovery rates of preadults, when comparing both infection experiments (see Fig. 2). However, the smaller chubs in the first experiment showed slightly lower mean recovery rates (17.9%) in comparison to the larger ones (27.0%) from the second experiment (Table 1, Fig. 2). The previous infection experiments with cystacanths of *Pomphorhynchus* sp. (see Siddall and Sures, 1998; Sures and Siddall, 1999, 2003; Sures *et al*., 2003; Ruchter, 2012; Le *et al*., 2016, 2018) showed a significantly higher overall establishment of *Pomphorhynchus* sp. in the final host in comparison to the preadults used in the current study (Kruskal–Wallis test, *P* < 0.05, see Fig. 2). The lowest recovery rates for cystacanths were recovered during the infection experiment of Sures and Siddall (1999) and the highest one was obtained by Sures and Siddall (2003) being 44.8 and 70.8%, respectively (Fig. 2).

## Discussion

Despite the fact that the acanthocephalans of the genus *Pomphorhynchus* occur at high prevalence and intensity in Ponto-Caspian gobies in both invasive and native distribution ranges (see Kvach and Skóra, 2006), the role of fish in parasite transmission is still unclear. Kennedy (2006) stated that acanthocephalans in accidental and paratenic hosts share the same morphological and developmental features. However, unlike those in accidental hosts, the acanthocephalans from a paratenic host can resume development if they are transferred to a suitable definitive host. Even adult acanthocephalans can be transmitted from one vertebrate host to another through a process known as post-cyclic transmission (Nickol, 2003; Kennedy, 2006), demonstrating the great flexibility of the acanthocephalan life-cycles (Perrot-Minnot *et al*., 2023). The fact that the recovered female acanthocephalans from the infected chubs in the present study harboured already mature eggs (spindle-like form) suggests that preadult individuals of *Pomphorhynchus* sp. that were collected from the body cavity of *N. melanostomus* can resume their further development to adults in an appropriate definitive fish host. Considering the high population density of gobies and their high infection rates with *Pomphorhynchus* spp. in newly invaded regions, it is likely that the gobies can contribute significantly to acanthocephalan's transmission and thus increase the infections levels in native host populations (amphipods and fish). It still remains unclear, which invasion scenario (spill back or spill over) regarding the *Pomphorhynchus* species in the Rhine River system is valid. Previous parasitological surveys on gobiids from Rhine River and other geographical regions have identified and reported the preadult acanthocephalans as *P. laevis* (see for example the publications of Ondračková *et al*., 2006, 2009, 2015) or *P. tereticollis* (e.g. Emde *et al*., 2012). However, according to the recent taxonomic studies on the genus *Pomphorhynchus* and the published sequences (see Reier *et al*., 2019), the individuals from gobiids in Rhine River (present study) should be *P. bosniacus*. Obviously, gobiids can host preadults of different species of the genus *Pomphorhynchus*, whereas for *P. laevis*, which is assumed as a native species in Central and Western Europe (see e.g. Kennedy, 2006; Médoc *et al*., 2011; Perrot-Minnot *et al*., 2019) a spill-back scenario *via* invasive gobiids might be possible. According to the original description of *P. bosniacus* (Kiskároly and Čanković, 1967) and recent studies (see Nedic *et al*., 2019; Reier *et al*., 2019), its geographical distribution is assumed to be restricted mainly to the Ponto-Caspian region and the Danube River system in particular. Thus, in the case of *P. bosniacus* a spill over scenario *via* gobiids might be possible, however, due to its missing invasion history it remains unknown if it was co-introduced in the Rhine River system with the invasion of gobiids (spill-over) or *via* other hosts (e.g. Ponto-Caspian amphipods). Additionally, the conflicting taxonomy within the genus *Pomphorhynchus* based on morphological and genetic identification does not allow a proper estimation of the geographical distribution of different *Pomphorhynchus* species (see e.g. the studies of Emde *et al*., 2012; Ondračková *et al*., 2015; Hohenadler *et al*., 2018*a*, 2018*b*, which were conducted in the lower Rhine River). This is especially true for *P. bosniacus*, whose identification and records were based solely on molecular data, with no morphological comparison to the type material of its original description was possible (see Reier *et al*., 2019).

The involvement of paratenic hosts is common in life cycles of acanthocephalans. However, this was mostly reported for species of the classes Archiacanthocephala and Eoacanthocephala (Schmidt, 1985; summarized also by Kennedy, 2006). Among the Palaeacanthocephala, to which species of the genus *Pomphorhynchus* belong, 4 genera have been reported so far (e.g. *Corynosoma*, *Leptorhynchoides, Andracantha, Bolbosoma*) that use paratenic hosts in order to bridge the trophic gap between intermediate and definitive hosts (see also Rocka, 2006). These acanthocephalans require mostly a piscivorous definitive host that do not regularly feed on crustaceans. However, this might be the case also for species of genus *Pomphorhynchus*, which possibly can also use an complementary transmission route *via* a paratenic host, as demonstrated by Médoc *et al*. (2011). The latter authors also reported very similar infection rates for *P. laevis* cysts obtained from the body cavity of minnows (*Phoxinus phoxinus*), which were used to experimentally infect chubs (15–23% *vs* 17–29% in present study). In contrast to gobies, minnows can serve as definitive host for *P. laevis*, however, the maturation rate of acanthocephalans in their gut is very low (see Kennedy, 1999).

Suitable definitive host for acanthocephalans of the genus *Pomphorhynchus* are usually cyprinids such as chub (*S. cephalus*) and common barbel (*B. barbus*) as well as salmonids (see e.g. Kennedy, 2006; Perrot-Minnot *et al*., 2019), however the latter rarely co-occur in the same habitats with gobies. Small fish might become one of the most important food items in the diet of large chubs or common barbel (Bašić *et al*., 2014; see also Kottelat and Freyhof, 2007), to which also gobiids are accounted (personal observation). The diet of chub is mostly habitat dependent, with a frequency of occurrence of fish in the diet of the chub of up to 8% in larger rivers (see e.g. Ünver and Erk'akan, 2011), which in some cases exceeds the weight percentage of all other food items (up to 95% as reported by (Losos *et al*., 1980). The common barbel commonly feeds during night upon benthic associated organisms having also access to prey items under larger stones (Vuković and Ivanović, 1971), where gobiids also hide to avoid predation. Benthic macroinvertebrates, small fish and fish eggs make up the majority of the barbel's diet, with the latter being the most common (see e.g. Losos *et al*., 1980). Considering that gobies might be an essential part of the diet of piscivorous fish after they have become established in newly invaded habitats (reviewed by Grabowska *et al*., 2023), as reported for pikeperch from the newly colonized Kiel Canal (Thiel *et al*., 2014) or for pikeperch and perch from western Baltic Sea (Oesterwind *et al*., 2017), they are most likely also used as food source by different large cyprinids and might additionally contribute to the transmission of *Pomphorhynchus* species.

Adults of *Pomphorhynchus* that are established in the intestine of a suitable fish definitive host can survive the predation of their fish host and even establish in the intestine of the predator. For example Kennedy (1999) investigated such a postcycling transmission of *P. laevis* by demonstrating the transfer of specimens from one definitive host to another. As the acanthocephalan's proboscis and bulb is commonly surrounded by fibrous tissue in the gut and body cavity of the definitive host, only non-mature adults of *Pomphorhynchus* sp. can survive the transfer and continue to mature and reproduce in the new fish host. The proboscis and bulbus of preadults obtained from the body cavity of gobiids were not encapsulated and remained intact, thereby being able to be used for establishment in the gut of an appropriate definitive host. However, most individuals were surrounded entirely by a fibrous capsule with variable thickness, whereas the preadults in the thick-walled cysts appeared less vital than the others with thin-walled, or without any cyst (personal observation). Prolonged residence in the body cavity of the gobies is likely to reduce the viability of the acanthocephalans, finally might lead to an inactivation (reduced infectiveness) and even death. In some cases, the parasites inside the thick-walled cysts appeared almost completely degenerated, which presumably was the result from the interaction with the host immune system. Similarly to the observations of Kennedy (1999), the preadult acanthocephalans in gobies can remain infectious probably only for a short period before they are surrounded with thick fibrous layer and thus become weakened/inactivated. Furthermore, Dezfuli *et al*. (2011) studied the fate of extraintestinal immature *Pomphorhynchus* sp. (preadults) encapsulated in the mesenteries and peritoneum of small sheatfish (*Silurus glanis*) that appeared to be similar to the ones found in gobiids. They found that the cyst wall consisted of 2 distinct layers with an outer one containing collagen fibres infiltrated with mast cells and an inner one, which was in direct contact with the parasite's tegument and comprised a large number of mast cells. Some of the latter were even located directly on the acanthocephalan tegument. As the mast cells were found to be the most common immune cells at attachment sites of *Pomphorhynchus* sp. and in the cyst wall of the extraintestinal preadults (Dezfuli *et al*., 2011) it can be assumed that they are responsible for the inactivation and destruction of parasites as suggested by Murray *et al*. (2007). Therefore, depending on the duration of the interaction between the parasites and the fish immune system, the acanthocephalans in gobies and other paratenic hosts can exhibit different fitness and capability to infect further fish hosts after predation. This would also explain the differences between the recovery rates of acanthocephalans obtained from the gobies (preadults) and those taken directly from the crustacean intermediate host (cystacanths) observed in the previous infection experiments (Siddall and Sures, 1998; Sures and Siddall, 1999, 2003; Sures *et al*., 2003; Ruchter, 2012; Le *et al*., 2016, 2018). The vitality and infectiveness of the cystacanths in amphipods appears to be less affected over time, due to the lower complexity of the invertebrate immune response (e.g. Dezfuli *et al*., 1992, 2008) in comparison to those of vertebrates (e.g. for fish Dezfuli *et al*., 2011) as well as the immune depression induced by cystacanths in their amphipod host (Cornet *et al*., 2009).

## Conclusions

The outcome of this study revealed that preadults of *Pomphorhynchus* sp. that occur in the body cavity of gobiids remain infectious and are able to resume their development in an appropriate fish definitive host. Accordingly, gobies and *N. melanostomus* in particular, might contribute to the transmission success of *P. bosniacus*, which could lead to a spill back to the local hosts in newly invaded habitats if the gobies are preyed by chub or barbel. However, due to the high abundance of gobies within the local fish community, a dilution-effect scenario might also occur, if they are not favoured by the definitive host as a prey item (effect on population level) or in the case that a longer interaction between the immune system of gobies and preadults lead to a lower infectiveness of the latter (effect on individual level). Therefore, further studies are required in order to extrapolate the findings of the current infection experiment to field conditions in Rhine River system. As no coinfection with other closely related species such as *P. tereticollis* was detected in the gobies in the present study, the role of gobiids in the transmission of other species of the genus needs to be further investigated. This might shed a light on interspecific competition between the species within the *Pomphorhynchus* genus occurring in the Rhine River system and thus reveal one of the mechanisms how *P. tereticollis* was outcompeted over time, as suggested by Hohenadler *et al*. (2018*b*).

## Data Availability

Data available on request from the authors.
